# Bone marrow stimulation for talar osteochondral lesions at long-term follow-up shows a high sports participation though a decrease in clinical outcomes over time

**DOI:** 10.1007/s00167-020-06250-8

**Published:** 2020-09-12

**Authors:** Kaj T. A. Lambers, Jari Dahmen, J. Nienke Altink, Mikel L. Reilingh, Christiaan J. A. van Bergen, Gino M. M. J. Kerkhoffs

**Affiliations:** 1 Department of Orthopaedic Surgery, Academic Medical Center, Amsterdam Movement Sciences, Amsterdam UMC, University of Amsterdam, Meibergdreef 9, Amsterdam, 1105 AZ The Netherlands; 2 Academic Center for Evidence Based Sports Medicine (ACES), Amsterdam, The Netherlands; 3 Amsterdam Collaboration for Health and Safety in Sports (ACHSS), AMC/VUmc IOC Research Center, Amsterdam, The Netherlands; 4 Department of Orthopedic Surgery, Amphia Hospital, Breda, The Netherlands; 5 Department of Orthopedic Surgery, Albert Schweitzer Hospital, Dordrecht, The Netherlands

**Keywords:** Osteochondral defect, Ankle, Bone marrow stimulation, Return to sport

## Abstract

**Purpose:**

Although bone marrow stimulation (BMS) as a treatment for osteochondral lesions of the talus (OCLT) shows high rates of sport resumption at short-term follow-up, it is unclear whether the sports activity is still possible at longer follow-up. The purpose of this study was, therefore, to evaluate sports activity after arthroscopic BMS at long-term follow-up.

**Methods:**

Sixty patients included in a previously published randomized-controlled trial were analyzed in the present study. All patients had undergone arthroscopic debridement and BMS for OCLT. Return to sports, level, and type were assessed in the first year post-operative and at final follow-up. Secondary outcome measures were assessed by standardized questionnaires with use of numeric rating scales for pain and satisfaction and the Foot and Ankle Outcome Score (FAOS).

**Results:**

The mean follow-up was 6.4 years (SD ± 1.1 years). The mean level of activity measured with the AAS was 6.2 pre-injury and 3.4 post-injury. It increased to 5.2 at 1 year after surgery and was 5.8 at final follow-up. At final follow-up, 54 patients (90%) participated in 16 different sports. Thirty-three patients (53%) indicated they returned to play sport at their pre-injury level. Twenty patients (33%) were not able to obtain their pre-injury level of sport because of ankle problems and eight other patients (13%) because of other reasons. Mean NRS for pain during rest was 2.7 pre-operative, 1.1 at 1 year, and 1.0 at final follow-up. Mean NRS during activity changed from 7.9 to 3.7 to 4.4, respectively. The FAOS scores improved at 1 year follow-up, but all subscores significantly decreased at final follow-up.

**Conclusion:**

At long-term follow-up (mean 6.4 years) after BMS for OCLT, 90% of patients still participate in sports activities, of whom 53% at pre-injury level. The AAS of the patients participating in sports remains similar pre-injury and post-operatively at final follow-up. A decrease over time in clinical outcomes was, however, seen when the follow-up scores at 1 year post-operatively were compared with the final follow-up.

**Level of evidence:**

Level II.

**Electronic supplementary material:**

The online version of this article (10.1007/s00167-020-06250-8) contains supplementary material, which is available to authorized users.

## Introduction

Osteochondral lesions of the talus (OCLT) are lesions to the subchondral bone and the overlying cartilage layer. These injuries have a high association with inversion injuries and ankle fractures [9, 21, 29]. Small (< 15 mm in diameter) lesions are initially treated with conservative treatment; however, in case of persistence of complaints, bone marrow stimulation (BMS) can provide good clinical outcomes with a success rate of 82% [2, 3]. Large primary defects that are amenable to fixation should be fixed whenever possible [10, 13]. In case of failure of primary surgical treatment, surgical treatment through an (osteo)chondral replacement strategy is indicated [9, 14].

As OCLTs are highly frequently seen in the athletic population, it is necessary to put specific emphasis on outcomes of treatment in athletes [23]. A recent systematic review by Steman et al. showed that return to sport (RTS) rates decreased when solely considering return to pre-injury level of sports [25]. Specifically considering BMS, in their review, the rate of return to any level of sports was 88%, and return to pre-injury level of sports 79%. Another recent review of the literature by Hurley et al. showed a rate of return to play as high as 86.8% after BMS with a mean time to return to play of 4.5 months [8]. In other research being conducted by Ramponi et al. [18], an RTS rate of 77% was found. None of these reviews, however, could provide adequate information on sports-related outcomes over time, as there is little literature on these outcomes on the long term. One retrospective study by van Eekeren found a return to sport rate of 76% after a median of 118 months [28]. Interestingly, the authors showed that the activity level seemed to decrease at long-term follow-up [28].

In most studies, it is often unclear whether authors reported on return to pre-injury level or return to any associated level of sports. Furthermore, there is a significant deficiency reported in rehabilitation protocols [8, 27]. Especially, the effect of BMS on return to sport after a longer period of time is scarcely reported in the literature. Bone marrow stimulation leads to the formation of fibrocartilage with an inferior subchondral bone plate as compared to original hyaline cartilage [16]. To date, it remains unclear whether this tissue enables high-level sports participation over time at the long term. The purpose of this study was, therefore, to prospectively evaluate return to sport and level of sport activity in patients treated with arthroscopic debridement and bone marrow stimulation and its persistence over time. It was hypothesized that return to sport rate and functional outcome would decrease over time.

## Materials and methods

**Fig. 1:**
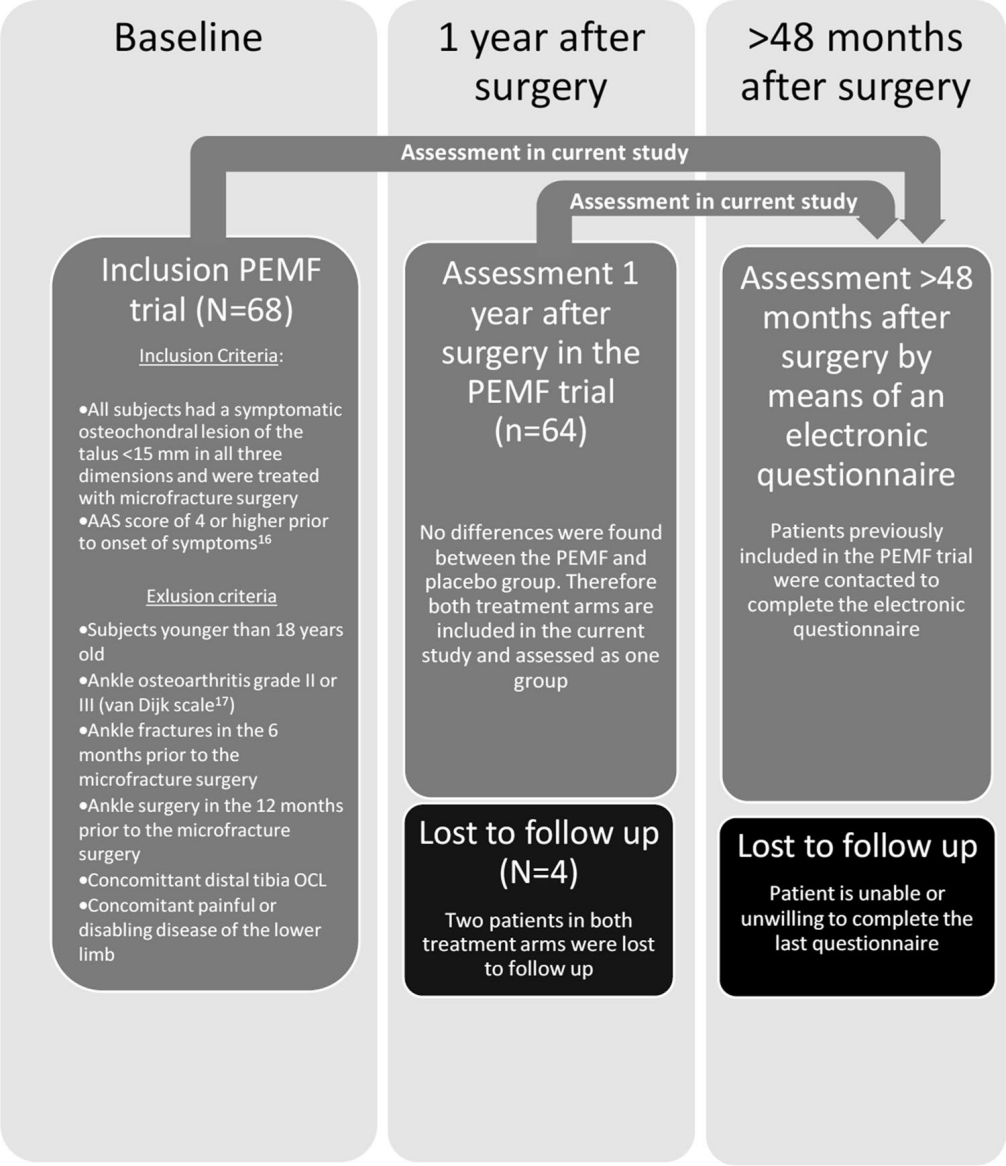
Study design

The study has been approved by the local medical ethics committee at the University of Amsterdam and was performed in accordance with the current medical ethical standards (Declaration of Helsinki, MEC 08/326). Written informed consent for participation was obtained from every patient. We included the patient cohort of Reilingh et al. and adhered to the inclusion and exclusion criteria of this previously published study (Fig. 1).

### Population

Patients included in a previously trial, the Pulsed Electro Magnetic Fields (PEMF) trial, were analyzed in the present study [19]. In that study, patients had undergone arthroscopic debridement and bone marrow stimulation alone in the control group, and patients included in the experimental group had undergone the same surgical treatment, but were treated post-operatively with pulsed electromagnetic fields (PEMF). Detailed information on the surgical procedure and post-operative rehabilitation in the PEMF trial have been previously described by Reilingh et al. [19]. Since the onset of the index trial, all patients have been followed up in a prospective manner by the institution. As the RCT by Reilingh et al. [19] concluded that PEMF did not lead to significant functional, sports nor radiological differences, we included both treatment arms as one treatment group in the present study.

### Postoperative rehabilitation

Patients followed a protocol-based rehabilitation program post-operatively and were guided by a physiotherapist. The patient was allowed to progress to full weight-bearing in 6 weeks. During this 6-week period, active nonweight-bearing and partial weight-bearing sagittal range of motion exercises were encouraged. After this period, resumption of sports was permitted as tolerated [27].

### Outcome measures

Clinical outcome assessment of the patients was performed by reaching out to the patients, requesting them to fill out an online questionnaire via the CASTOR^©^ portal. To reach all patients from the previous PEMF trial, consisting of a cohort of 68 patients, a personalized email was sent out to the potential subjects of the study [19]. In case of no response from the study subjects, two personalized reminder emails were sent. If the questionnaire still was incomplete, the authors of the present study sought contact by means of reaching out to the patients via telephone.

Through the online post-operative questionnaire, return to sports was assessed, including type and level of sports. Concerning sports activities, the Ankle Activity Scale (AAS) was used [7]. The AAS is a survey containing a high number of sports (*n* = 53), 3 working activities, and 4 general activities [7]. Scoring zero points on the scale indicates the lowest activity and ten points indicate the highest activity. The AAS was developed by Halasi et al. with the purpose to develop a widely usable, ankle specific activity scoring system that includes most internationally known sports [7]. The score was based on a previously developed activity score for knee injuries, named the Tegner score [1]. Furthermore, secondary outcome measured was assessed by means of the numeric rating scale 0–10 (NRS) for pain (at rest and during activity) [6], the Foot and Ankle Outcome Score (FAOS) [20, 24], work activities, and the subjective satisfaction concerning the surgery. Patients were requested to fill out whether they suffered from any other musculoskeletal injuries from the time of surgery till the moment of filling out the online questionnaire. The NRS is an 11-point scale, representing the spectrum of no pain (0 points) to the worst pain imaginable (10 points) [6].

The sport outcomes, the AAS, the NRS scales (at rest and when running), the FAOS, and return to work had also been assessed in the previous study by Reilingh et al. [19] both pre-operatively and 1 year post-operatively which, therefore, facilitated a formal comparison in changes over time.

### Statistical analysis

All analyses were done using Statistical Packages for Social Sciences (SPSS 24.0 Inc, Chicago, IL, USA). To minimize the risk of attrition bias, we included all patients with a completed electronic questionnaire at final follow-up in the statistical analyses. This includes the patients who underwent a reoperation. No additional sample size calculation was performed as this study was a follow-up study of a previously published study. Detailed information on the sample size calculation is described in this previous study [19]. Descriptive statistics of categorical variables were presented as frequencies with percentages per category. Differences in FAOS scores, NRS of pain, and AAS at final follow-up were assessed between patients in the two different original treatment groups (PEMF and Placebo) by means of an independent samples t-test.

Descriptive statistics of continuous variables were calculated as means ± standard deviations or median and ranges in case of a skewed distribution. Categorical variables were presented as frequencies with percentages. Differences of the continuous outcome variables were assessed between baseline and final follow-up and between 1 year follow-up and final follow-up. In case of normally distributed data, a paired t-test was used; in case of skewed data, a Wilcoxon signed-rank test was used. The comparisons with *p* < 0.05 were considered to be statistically significant.

## Results

Four patients of the 64 (6%) were lost to follow up and could not be reached. The other 60 patients were available for follow-up. No patients declined to participate. The mean post-operative follow-up was at 6.4 years after surgery (SD ± 1.1 years). Mean age of the patients at follow-up was 39 years (SD ± 8.5 years).

### Sub-analysis original group

A sub-analysis of the original groups (the PEMF group and the placebo group) showed no significant difference at any of the outcome measures (AAS, NRS pain rest, NRS pain activity, FAOS other symptoms, FAOS pain, FAOS ADL, FAOS sport, and FAOS QOL). Data could thus be interpreted as one group.

### Outcome

#### Sports outcomes

**Fig. 2:**
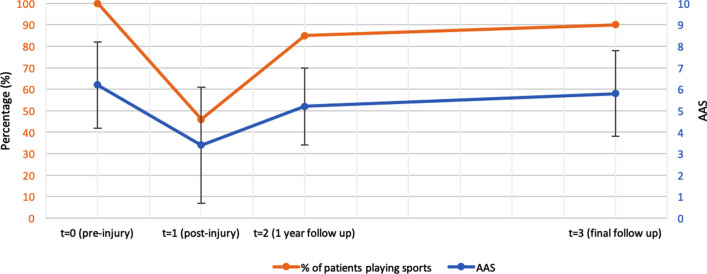
Percentage of patients playing sports and Ankle Activity Score (AAS) over time

Of all included patients, all participated in sports before injury of which 6 on a professional level, 29 on a competitive level, and 25 on a recreational level. The median pre-injury ankle activity score of the 60 included patients was 6.2 (range 4–8). After onset of complaints pre-operatively, only 27 patients were still participating in sports of which 2 on a professional level, 6 competitive, and 19 recreational. The other 33 were not able to participate in sports anymore. The AAS decreased to 3.4 post-injury. After surgery, the median time to return to sports of the 60 included patients was 18 weeks. At 1 year after surgery, 85% of patients returned to playing sport and the AAS increased to 5.2. At final follow-up, 90% of patients participated in sports. These 54 patients participated in 16 different sports, some participating in more than one sport. Most played sport was soccer (16 patients) followed by running (14 patients), fitness (12 patients), cycling/mountainbiking (9 patients), and tennis (7 patients). Of all patients playing sports at follow-up, 35 patients played at a recreational level and 18 patients played at a competitive level. One patient played table tennis on a professional level for years after surgery. At final follow-up, the median current level of activity of all the patients measured with the AAS was 5.8 (range 1–9) (Fig. 2). If we excluded the 6 patients not playing sports anymore, the median AAS of the 54 patients still playing sports was 6.0 (range 3–9).

At time of final follow-up, 32 patients (53%) indicated that they returned to play sport at their pre-injury level. Twenty patients (33%) were not able to obtain their pre-injury level of sport because of ankle problems despite the operation. Another eight patients (13%) indicated that they were not able to play sport at their pre-injury level because of different reasons than their ankle.

#### Reoperations

Three patients underwent 4 reoperations in total. One patient received an ankle arthrodesis at 6 years after the original surgery because of progressive osteoarthritis and experienced pain. One patient received a reoperation due to posterior impingement 15 months after the original surgery. This and another patient also underwent an adjacent procedure in another hospital.

#### Additional injuries

Twenty-six patients experienced one or more additional injuries, e.g., back pain or spine injuries (5 patients), anterior cruciate ligament tears (4 patients), other knee problems (5 patients), and achilles tendon problems (3 patients).

#### Work-related outcomes

Fifty-eight patients resumed to their pre-injury work. One patient indicated that he worked less due to persisting ankle problems. Seven worked less due to other reasons. Four patients reported to be working more because of a decrease in ankle problems.

#### Clinical outcomes

**Fig. 3:**
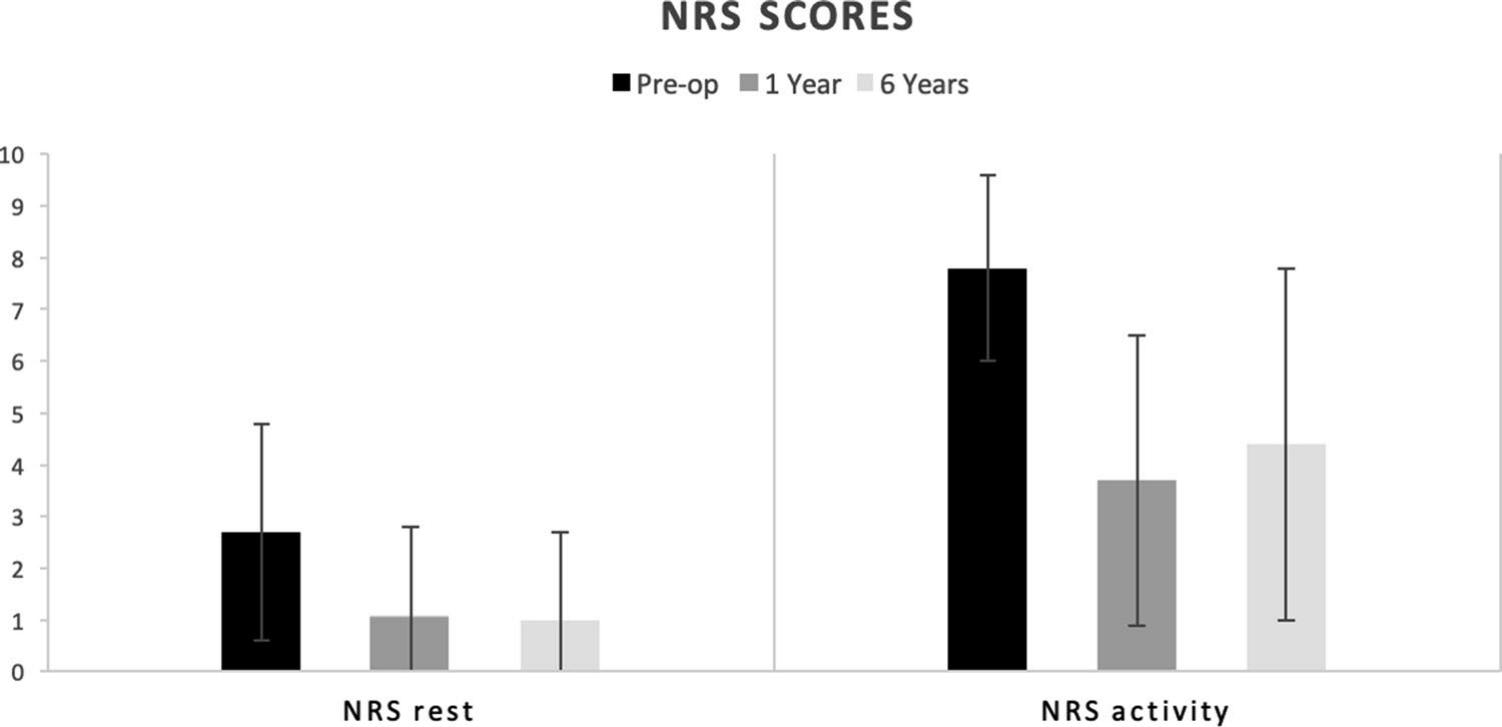
NRS subscores (mean scores and SD) at different follow-up moments (pre-operatively, at 1 year follow-up and at final follow-up at 6.4 years)

**Fig. 4:**
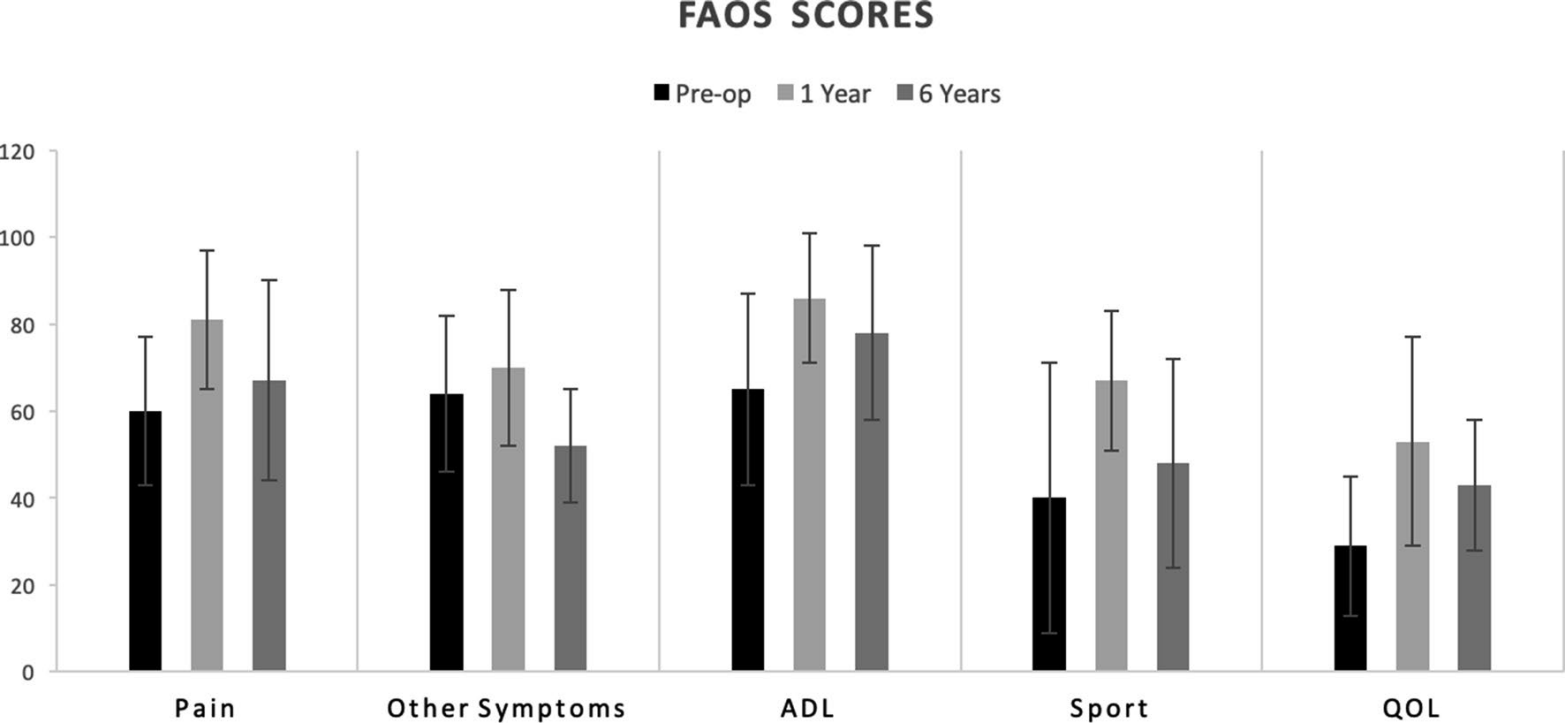
FAOS subscores (mean scores and SD) at different follow-up moments (pre-operatively, at 1 year follow-up and at final follow-up at 6.4 years). *ADL* activities of daily living, *QOL* quality of life

The clinical outcomes (NRS and FAOS scores) are presented in Figs. 3 and 4. Between the pre-operative scores and final follow-up, the NRS pain during rest decreased from 2.7 to 1.0 (*p* < 0.01) and the NRS pain during activity from 7.9 to 4.3 (*p* < 0.01). The FAOS other symptoms decreased from 64 to 52 (*p* < 0.01), the FAOS pain increased from 61 to 67 (*p* = 0.01), the FAOS ADL from 66 to 79 (*p* < 0.01), the FAOS sport from 41 to 48 (n.s.), and the FAOS QOL from 29 to 44 (*p* < 0.01).

Between 1 year and final follow-up, the NRS did not change significantly with the NRS during rest from 1.1 to 1.0 (n.s.) and the NRS activity from 3.7 to 4.3 (n.s.). All the FAOS scores, however, significantly decreased. The FAOS other symptoms decreased from 70.6 to 52 (*p* < 0.01), the FAOS pain from 82 to 67 (*p* < 0.01), the FAOS ADL from 86 to 78 (*p* = 0.02), the FAOS sport from 67 to 48 (*p* < 0.01), and the FAOS QOL from 54 to 44 (*p* < 0.01).

### Patient satisfaction

In total, 26% of patients were very satisfied with the procedure, 43% of patients said they were satisfied, 17% were neutral, and 15% were unsatisfied. No patients reported to be very unsatisfied. 65% indicated that they would undergo the same procedure if they had to choose again, 15% would not, and 20% were neutral on this subject. Patients rated the end result with a mean of seven in a score ranging from 0–10 (SD = 2).

## Discussion

The most important finding of the present study was that arthroscopic BMS in OCLT results in 90% of return to sports/physical activity with 53% being able to return to their pre-injury level of sport at a mean follow-up of 6.4 years. This percentage of sports participation is comparable with follow-up at 1 year post-operatively. The AAS of the patients participating in sports remains similar pre-injury and post-operatively at final follow-up. However, a decrease over time in clinical outcomes were seen when we compare the FAOS follow-up scores at 1 year post-operatively with the final follow-up.

Return to sports is important in most young and active patients and of course of uttermost importance in professional athletes. The sustainability of the repaired cartilage is essential in this group to remain active at the highest level. High reported RTS rates at pre-injury level have been reported after BMS in the ankle of up to 94% in elite athletes after a mean follow-up of 3.6 years [22]. Similar percentages are found when looked at autologous bone grafting techniques with a slightly longer RTS time (19.6 ± 5.9 vs 15.1 ± 4 weeks, respectively) [5, 21, 22]. These numbers are much higher than the RTS to pre-injury level which we found being 53% at final follow-up. Multiple explanations can be imagined. Of course, general aging can be of a factor when a lessened level of sport is found. Furthermore, the follow-up time has a negative influence in sport participation, because the quality of fibrocartilage may decrease in time and progression of osteoarthritis may increase in time. Other reasons for decrease of level of activity can also be found in problems other than the ankle, but we tried to overcome this by asking why patients were not able to play sports at their pre-injury level anymore. This still left 33% of them not being able to perform on pre-injury level because of problems with their ankle. Another possibility why the found percentage at the high active patients is higher might be because of the competitive nature of elite athletes and their high drive to perform on the highest possible level.

With multiple techniques being available, bone marrow stimulation still is worldwide the most performed intervention for both primary and secondary osteochondral lesions of the talus [3, 14]. It is a relative easy and low-demanding technique with low costs compared to the costlier transplantation or implantation techniques. It is also still the most studied technique showing good-to-excellent results in general [3, 11, 14]. The outcome regarding RTS time and rates after bone marrow stimulation is less described. A few studies focusing specifically on high demanding athletes previously reported good results and a review by Hurley et al. showed a high reported return to play in general after BMS [8, 21, 22]. However, only 14% of the included studies in this review adequately reported the rate of return to sports and they conclude that there is little literature on long-term outcome of BMS for OCLTs in the athletic population [8]. With the larger studies that do describe sports outcome at long-term follow-up being retrospective in nature, we can conclude that there is still a lack of prospective studies collecting data about the long-term outcome [8, 21–23, 26, 28]. This while more causes for concern are shown in the long term. These concerns are regarding the sustainability of this technique with second-look arthroscopies showing lack of infill at the lesion or the inability to return to the same level of sport [15, 23]. Deterioration of the repaired cartilage was noted in 35% of patients at 5 years second-look arthroscopy and a progress of osteoarthritis by one grade on standard radiographs was reported in 33% of patients with a mean follow-up of 141 months [4, 26]. Long-term (prospective) research is scarce especially concerning sport activities. The aim of this study was, therefore, to report on these outcomes after BMS.

The present study has to be interpreted in light of its strengths and weaknesses. Since four patients were lost to follow up from a group of 64 patients, the lost to follow-up percentage is calculated to be 6%. An acceptable lost to follow up, however, according to the Center for Evidence Based Medicine. Furthermore, since the lost to follow up presumable is missing at random after a mean follow-up period of more than 6 years, we think that it is an adequate follow-up and representative study group [12]. Another limitation is that not all outcome measures were used at final follow-up compared to baseline and 1 year after surgery. In contrast to the pre-operative and short-term follow-up moments, the AOFAS hindfoot score was not conducted at final follow-up. The AOFAS clinical rating systems have insufficient reliability and validity, and would, therefore, provide limited additional information, while patient burden would have been significantly increased as this is a clinician based score [17]. Finally, at final follow-up, no radiological examination by means of a CT scan has been performed which could have been interesting from a research point of view.

The results of this study can be used to inform patients, specifically active patients, about expectations around long-term sports outcome after bone marrow stimulation. It also shows that although there is a high return to sport participation, there is still room left for improvement, since outcome scores decreased over time. Possible further research could focus on sport outcome after treatment types that are more based around the preservation of hyaline cartilage such as transplantation or fixation techniques.

## Conclusion

Arthroscopic BMS in OCLT results in 90% of return to sports/physical activity with 53% being able to return to their pre-injury level of sport at a mean follow-up of 6.4 years. The percentage of sports participation is comparable with follow-up at 1 year post-operatively. The AAS of the patients participating in sports remains similar pre-injury and post-operatively at final follow-up. A decrease over time in clinical outcomes was seen at final follow-up.

## Electronic supplementary material

Below is the link to the electronic supplementary material.

Supplementary file1 (DOCX 70 kb)
