# Supplementary material for: Bone marrow stimulation for talar osteochondral lesions at long-term follow-up shows a high sports participation though a decrease in clinical outcomes over time
Source: Knee Surg Sports Traumatol Arthrosc. 2020 Sep 12;29(5):1562–9. doi: 10.1007/s00167-020-06250-8 (PMC8038982; doi:10.1007/s00167-020-06250-8)
Supplement: Supplementary file 1 — Supplementary file1 (DOCX 70 kb) [file 167_2020_6250_MOESM1_ESM.docx]

**APPENDIX**

Question included in the online questionnaire via CASTOR:

- FAOS
- NRS
  - In rest
  - During activity
- Satisfaction about the surgery
  - Would you undergo the surgery again (yes/no/neutral)
  - How satisfied are you with the surgery (very satisfied, satisfied, neutral, unsatisfied, highly unsatisfied)
  - What would be the satisfaction grade concerning the surgery (1-10)
- Return to sports?
  - Did you perform sports after the surgery? (Yes/no)
  - What kind of sports
  - At what date did you perform sports after the surgery again?
  - At what level? (professionally/competitively/recreatively)
  - Would you be able to choose the best option about the sports level (I perform sports more often now than prior to the surgery because of less ankle complaints, I perform more sports than prior to the surgery because of other reasons,I perform sports just as much as before the surgery, I perform less sports because of ankle complaints, I perform less sports because of other reasons than my ankle)
- Did you have other complaints or injuries after the surgery up to the follow-up with questionnaires
  - yes / no
  - What kind of injury did you have
- return to work
  - After the surgery (yes/no)
  - At what date did you return to work
  - Would you be able to choose the best option about the work level (I perform more work activities now than prior to the surgery because of less ankle complaints, I perform more work than prior to the surgery because of other reasons,I perform work just as much as before the surgery, I work less because of ankle complaints, I work less because of other reasons than my ankle)
